# Predictions of Native American Population Structure Using Linguistic Covariates in a Hidden Regression Framework

**DOI:** 10.1371/journal.pone.0016227

**Published:** 2011-01-31

**Authors:** Flora Jay, Olivier François, Michael G. B. Blum

**Affiliations:** Université Joseph Fourier, Grenoble, Centre National de la Recherche Scientifique, Laboratoire des Techniques de l'Ingénierie Médicale et de la Complexité, Equipe Biologie Computationnelle et Mathématique, Faculté de Médecine, La Tronche, France; Aarhus University, Denmark

## Abstract

**Background:**

The mainland of the Americas is home to a remarkable diversity of languages, and the relationships between genes and languages have attracted considerable attention in the past. Here we investigate to which extent geography and languages can predict the genetic structure of Native American populations.

**Methodology/Principal Findings:**

Our approach is based on a Bayesian latent cluster regression model in which cluster membership is explained by geographic and linguistic covariates. After correcting for geographic effects, we find that the inclusion of linguistic information improves the prediction of individual membership to genetic clusters. We further compare the predictive power of Greenberg's and *The Ethnologue* classifications of Amerindian languages. We report that *The Ethnologue* classification provides a better genetic proxy than Greenberg's classification at the stock and at the group levels. Although high predictive values can be achieved from *The Ethnologue* classification, we nevertheless emphasize that Choco, Chibchan and Tupi linguistic families do not exhibit a univocal correspondence with genetic clusters.

**Conclusions/Significance:**

The Bayesian latent class regression model described here is efficient at predicting population genetic structure using geographic and linguistic information in Native American populations.

## Introduction

Comparing genetic and linguistic data provides information about various aspects of American prehistory, the process by which the Americas were originally colonized [1] or migration across linguistic barriers [2]. In addition to anthropological applications, evaluating the relationships between genes and languages has potential biomedical applications since language could be used as a *proxy* for genetic ancestry in various epidemiological contexts [3], [4].

Previous analyses comparing genetic to linguistic differentiation in the Americas yielded equivocal results. Cavalli-Sforza *et al.* [5] reported that, prior to the publication of their book, three of seven studies supported congruence between genes and languages [6]–[12]. At that time, Ward *et al.* [13] found that rates of linguistic diversification are faster that rates of genetic differentiation in mtDNA, and concluded that there is little congruence between linguistic and genetic relationships in the Americas. In more recent studies also using mtDNA, the hypothesis that language classifications reflect the genetic structure of Native American populations was also rejected [2], [14]. Lastly, an analysis of autosomal microsatellite markers in 28 Native American populations from the Human Genome Diversity Panel (HGDP) provided a qualitative correspondence between linguistic and genetic groupings [15]. However, tests of correlation were not significant for these data.

To investigate the relationships between genes and languages, the previous studies made use of tree-based or distance-based methods. Hunley and Long [2] and Hunley *et al.* [14] applied a test of treeness developed by Cavalli-Sforza and Piazza [16] to decide if a matrix of genetic distances is compatible with a language tree. These authors dealt with various hierarchical classifications of American languages, and they found that none of them were consistent with the mitochondrial genetic distances. Adopting another approach, Cavalli-Sforza *et al.* [17] found a high degree of association between linguistic and genetic trees using a consistency index. Alternatively, the association of genes and languages can be assessed by Mantel tests [18]. Mantel tests are used to reject the absence of correlation between a matrix of genetic distances and a matrix of linguistic distances, and do not require reconstructing population trees. Since a spurious association between genetic and linguistic distances may be detected when geography is not accounted for, more elaborate procedures called partial Mantel tests can be applied in order to control for geography [19]. Partial Mantel tests were applied to the HGDP and did not provide strong evidence of association in Native American populations [15].

By definition, the results obtained from tree-based and distance-based methods are influenced by specific choices of tree reconstruction methods or particular genetic and linguistic distances. The validity of population trees depends on the reliability of their reconstruction method and on the hypothesis that genetic differentiation results from population fission. Whereas trees are well-suited for describing evolutionary relationships of non-recombining sequences like mtDNA, they may be sensitive to distortion due to gene flow between populations when nuclear data are analyzed [20]. In addition, we still lack an evolutionary tree for languages as linguists have not yet reached a clear consensus on their classification [21], and even questioned the validity of branching trees as an adequate representation of linguistic patterns of divergence [22]. Finally, there are several pairwise measures of population differentiation or of linguistic divergence, and the choice of a specific measure can have a significant impact on Mantel tests [23]. Linguistic distances can, for instance, be based on a hierarchical linguistic classification [24], or they can be directly derived from structural linguistic features such as aspects of sound systems and grammar [25], [26].

In this study, we introduce a novel method for investigating the relationships between genes and languages that avoids genetic and linguistic distances as well as tree reconstruction methods. We consider Bayesian *latent class regression* models [27] where we regress the unobserved genetic structure on linguistic and geographic variables. The principle of the method is to group individuals into genetic clusters at the same time as their latent cluster labels are regressed. To evaluate the predictive capacity of different sets of linguistic and geographic covariates, we also propose procedures of variable selection. Using this approach, the following questions are addressed. To what extent can geographic or linguistic origin explain individual membership to genetic clusters? Do languages contribute to a better prediction of cluster membership than geography alone? Among the classifications of Native American languages that have been proposed by linguists [28], [29], which one is the best predictor of population genetic structure? Although some of these questions have received considerable attention in the context of evolutionary trees or evolutionary distance comparisons [2], [23], [30], examining their answers from a latent class individual-based model is new and potentially highly informative.

## Methods

Several Bayesian model-based approaches have been proposed to assign individuals to genetic clusters [31]–[33]. To assess the effects of geographic and linguistic covariates on the assignment of individuals to genetic clusters, we considered a Bayesian latent class regression model [27], [34], [35]. This new model incorporates a hidden regression model within the framework proposed by Pritchard *et al.* [31] and implemented in the computer program structure.

### Bayesian model

Consider a genotypic data set, 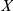, for a sample of 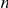 diploid individuals genotyped at 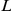 loci, and assume that there are 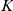 clusters, each of which is characterized by a set of allele frequencies at each locus. Let 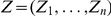 be the vector of cluster labels of each individual in the sample, and let 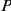 be the set of allele frequencies. In addition, assume that a set of covariates is measured for each individual, and stored in a design matrix, 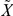. The covariates represent the geographic and linguistic information that is available to build predictors of the population genetic structure that is encoded in vector 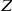. Regarding geography, predictors can be defined as linear or quadratic trend surfaces as proposed by Durand *et al.* [36]. Linear trend surfaces include two covariates, latitude and longitude, while quadratic surfaces also include squared and cross-product terms. Languages are coded as factors defined as binary dummy variables in the design matrix [37]. The factor levels will be dependent on the choice of the linguistic classifications considered further in this study. Remark that in regression models using factors, a linear constraint (or contrast) must be defined for identifiability reasons. In our study, we assumed that the sum of effects is null.

For algorithmic reasons, the latent regression model was implemented through a hidden *multinomial probit model* [38]. In the multinomial probit model, there are 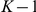 regression equations
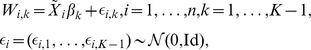
(1)each corresponding to a genetic cluster. The 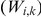 are “augmented” continuous variables defined for each individual and each cluster, 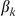 is a column vector of regression coefficients, and 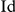 denotes the identity matrix. For each individual 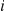, a cluster label 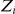 can be obtained from the augmented variables as follows
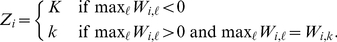
(2)


In the multinomial probit model the role of the clusters is not symmetric. The estimates of the regression coefficients are defined with respect to the 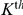 cluster, called the *reference cluster*.

Given the above latent class model framework, we used a Markov Chain Monte Carlo (MCMC) algorithm based on Gibbs sampling to compute the joint posterior distribution on individual cluster labels, regression coefficients and allele frequencies
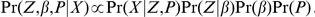



In this equation, the likelihood 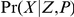 and the prior distribution on allele frequencies 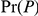 are computed in the same way as in the model without admixture of the program structure (equations (2) and (4) in [31]). The distribution 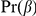 is a noninformative prior distribution (see Appendix A), and 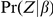 corresponds to the distribution of cluster labels obtained from the multinomial probit model. The algorithm was implemented in the software POPS, and is described in more details in Appendix A.

For each subset of covariates, we additionally computed a matrix of posterior predictive membership probabilities using a Monte Carlo method. To perform the computations, we simulated cluster labels from the generative model described in equation (1) and (2) where the regression coefficients are sampled from their posterior distribution. To display predicted and inferred membership probabilities graphically, we used barplot representations. In these graphics, each individual is represented by 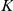 aligned colored segments, and the segment lengths are proportional to their estimated or predicted membership probabilities.

### Variable selection

To investigate whether a particular subset of covariates is a suitable proxy for genetic assignment, we used two distinct measures. Both measures are based on the posterior of regression coefficients and cluster labels. The first measure is a Pearson correlation coefficient, 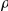. For a given subset of covariates, the ability of the model to predict genetic structure was evaluated by computing the correlation between the matrix of predicted membership coefficients and the matrix of estimated membership coefficients. The second measure is based on cross-validation, a technique used in the field of machine learning [39], [40] and for latent class models [41]. In our analyses, a 2-fold cross-validation was implemented. More specifically, we divided the genotypic data set, 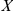, into two non-overlapping data sets containing complementary subsets of loci. We considered one of these data sets as the training set, 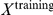, and the other one as the validation set, 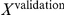. The rationale of the cross-validation approach is that the demographic processes that shaped population genetic structure have affected all loci across the genome. Thus the training and validation sets are exchangeable, as they provide the same amount of information about population structure. We performed 500 runs of the Gibbs sampling algorithm using the training set, and retained the 50 runs having reached the highest likelihood values. For each of the retained runs, a predictive score was computed by averaging the log-probability of the validation set over the posterior distribution given the training set
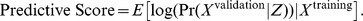



The computation of predictive scores is detailed in Appendix B. Another series of 50 scores was computed after exchanging the role of the validation and training sets, and a cross-validation score was obtained by averaging the resulting 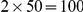 predictive scores.

### Simulated data

We ran a first series of simulations using the generating model of the program POPS. Assuming three clusters, cluster labels of 300 individuals were simulated using the following regression equations
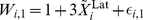
(3)

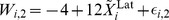
(4)where 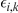 is a standard Gaussian noise. The interpretation of the above linear trend model is that latitude is the only variable that influences individual cluster labels. Biallelic genotypes were simulated at 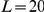, 40, 100 loci. Allele frequencies were dependent on the population of origin, and were equal to 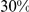 and 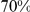, 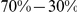 and 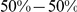 in each population respectively. We implemented four hidden regression models: one model without covariates, one with latitude, one with longitude and one with both covariates.

In the second series of simulations, we extended the model by including a factor with five levels representing five languages. The hidden regression equations were defined as
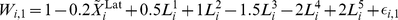
(5)

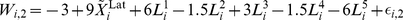
(6)where 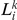 is equal to 1 if individual 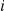 speaks the language 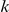 and is 0 otherwise. When running POPS to predict population genetic structure, we considered three linguistic classifications. The first classification contained five languages corresponding to the indicator variables used in the simulation. The second classification contained seven languages obtained after splitting the second and the third languages of the first classification into two sublanguages. The last classification contained three languages because we merged two pairs of unrelated languages from the first classification.

In the third series of experiments, we studied two previously published data sets simulated from a five-island model [42]. The simulated data represented one population structured into five subpopulations differentiated at 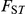 levels equal to 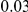 and 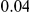. Five hundred individuals (100 per subpopulation) were simulated using allele frequency distributions across 10 codominant unlinked loci. Spatial coordinates were simulated using Gaussian distributions. The subpopulations were adjacent to each other and arranged on a ring. We ran POPS using the spatial coordinates of each individual as covariates. In addition, we introduced a spurious noisy covariate independent on the subpopulation of origin. We considered the models defined by all the possible inclusions of those three covariates (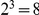 models). These data enabled us to compare the performances of POPS to other programs using spatial covariates [42]–[44].

### Native American data

We applied POPS to 512 Native American individuals from the Human Genome Diversity Panel (HGDP) data set [15]. Individuals from 28 populations were genotyped at 678 microsatellite loci. Fourteen Siberian individuals from the Tundra Nentsi population were also included in the study. In the regression models we considered three linguistic classifications. The first and second linguistic classifications corresponded to Greenberg's classification at the stock level and at the group level [28], [45]. The third linguistic classification was given by the website *The Ethnologue* (www.ethnologue.com) [29], [46]. The three linguistic classifications were encoded with factors having 8, 14 and 16 levels respectively (see ). To account for geography, all models included quadratic trend surfaces. The combinations of geographic and linguistic variables resulted in the following four latent cluster regression models. Model A included geographic information only. Models B-D included geographic and linguistic information: Model B used Greenberg's classification at the stock level (8 levels), Model C used Greenberg's classification at the group level (14 levels), and Model D used *The Ethnologue* classification at the family level (16 levels).

### MCMC parameters

For the simulated data, the runs of POPS used 2,000 sweeps with an initial burn-in period of 1,000 sweeps. For the human data, the runs used 5,000 sweeps with an initial burn-in period of 2,500 sweeps. These values ensured that the likelihoods stabilized around their stationary values. For the HGDP data and for each model, we ran a total of 500 MCMC runs. We retained the 50 runs with the largest likelihood values, and we averaged the resulting estimated and predicted membership coefficients using the computer program clumpp [47].

The number of clusters was set to 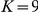 [15]. Among these nine clusters, there were eight Native American clusters plus the reference cluster. For Native American population samples, we chose the Siberian population (Tundra-Nentsi) to represent the reference group. Individuals in the reference cluster were not allowed to switch to other clusters during the MCMC runs.

## Results

### Simulation results

Using simulated data sets, we investigated whether including geographic and linguistic covariates can improve the estimation of membership probabilities or not, and we evaluated which subsets of variables best predict the estimated population genetic structure.

**Figure 1:**
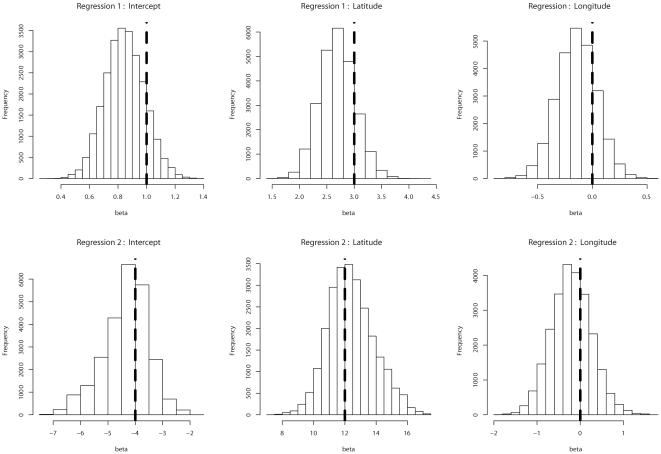
Posterior distributions of the regression coefficients for a data set simulated with the hidden regression model (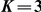). The dashed vertical lines correspond to the regression coefficients used for generating the data. Two spatial covariates (latitude and longitude) are included in the regression model but only the first one influences genetic structure.

**Figure 2:**
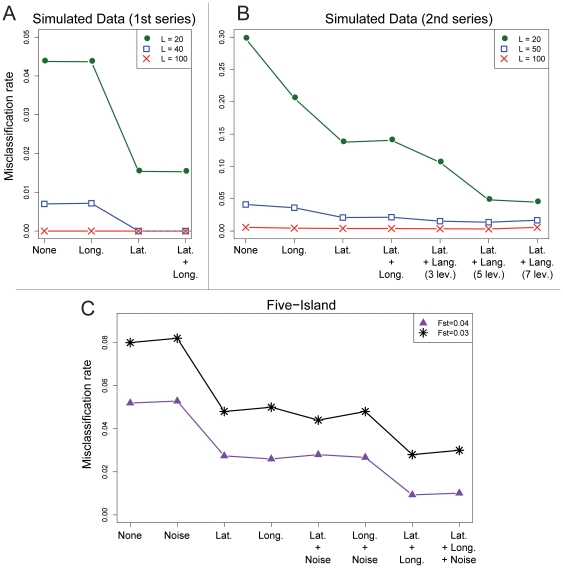
Misclassification rates for simulated data as a function of the covariates included in the clustering algorithm. A. The cluster memberships are influenced by latitude but not by longitude. B. The data are generated using latitude and a 5-level linguistic classification. C. The data are generated in a five-island model for which 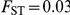 or 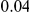.

For the simulations where latitude was influential (equations (3) and (4)), we found that the true values of the regression coefficients were close to the mode of the posterior distributions (Figure 1). The influence of each covariate was thus correctly ascertained by POPS when the data were generated under its underlying statistical model. To further evaluate if missing the true set of covariates modifies the inference and the prediction of membership coefficients, we evaluated the performances of POPS using various hidden regression models. For all models, the misclassification rates were less than 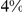. The upper bound was obtained under a model without covariates and for the smallest number of loci (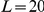, Figure 2A). The misclassification rates never increased when we included a spurious longitude variable. With 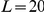 loci, the misclassification rate decreased to 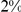 when the correct covariate (latitude) was used. With 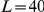 loci, the misclassification rates were less than 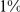 for all hidden models. All individuals were perfectly assigned to their population of origin when latitude was included. For 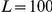, the misclassification rate was equal to 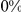 for all models. In the second series of simulations, linguistic covariates were added to the generating model (equations (5) and (6)). The misclassification rates were less than 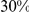, a value obtained for 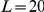 loci in a model without covariates (Figure 2B). With 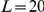, the misclassification rate decreased to 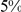 when including latitude and a linguistic variable with five levels. With 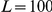 loci, the misclassification rate of the model without covariates was around 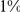. We conclude that when the data are generated from a hidden regression model, including covariates in POPS increases the performances of the program. This is particularly true when the number of loci is relatively small.

**Figure 3:**
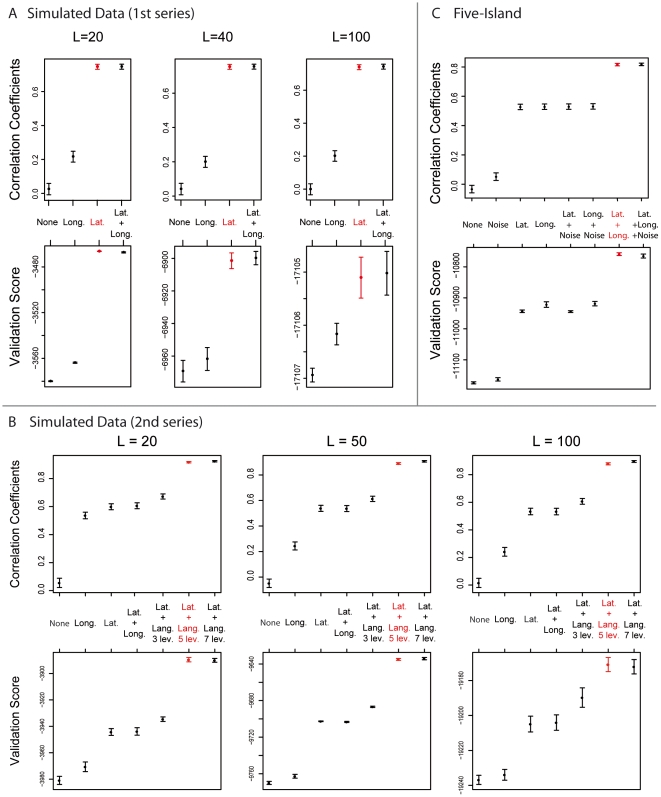
Variable selection for simulated data. The correlation coefficients 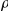 correspond to the correlations between the estimated and predicted membership probabilities. Confidence intervals of the correlation coefficients are estimated by assuming that the Fisher's transform 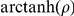 follows a Gaussian distribution [65]. The validation scores are estimated with the 2-fold cross-validation method. Their standard deviations are estimated by using a non-parametric bootstrap method. A. The cluster memberships are influenced by latitude but not by longitude. B. The data are generated using latitude and a 5-level linguistic classification. C. The data are generated in a five-island model for which 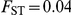.

Finally, we studied the variable selection criteria for the data where latitude was influential (equations (3) and (4)) as well as linguistic covariates (equations (5) and (6)). Whatever the number of loci we considered, the increase of the correlation coefficient was larger when including latitude rather than longitude in the regression model. Figure 3 shows that the correlation coefficient and the cross-validation score reach a plateau when the true predictors are included in the hidden regression model. This plateau was found when latitude was the sole determinant of genetic structure and when linguistic covariates had an additional contribution to genetic differentiation.

For the five-island data with a level of differentiation of 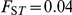, the misclassification rates were less than 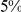 (Figure 2C). The worst performances were obtained for a model without covariates. When latitude (or longitude) was included in the hidden regression model, the misclassification rate decreased to 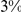. When both latitude and longitude were included in the model, the misclassification rate decreased to 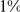. The addition of a spurious noisy covariate did not impact the performance of the program. Regarding variable selection, Figure 3C shows that the correlation coefficients and the validation scores reach a plateau when longitude and latitude are included in the hidden regression model. For the five-island data with a level of differentiation of 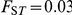, a model including latitude and longitude was also selected. In this case, the misclassification rate was equal to 2.8%. For these data, POPS compared favorably to the spatial versions of BAPS (misclassification rate = 3.9%) and TESS (misclassification rate = 4.4%) [42]–[44].

### Native American HGDP data

To investigate the relationships between geography, languages and genes in Native American populations, we applied POPS to a multilocus genotype data set including 512 individuals from the HGDP. We compared the posterior membership coefficients predicted by four different models that use distinct linguistic classifications and we computed two variable selection criteria in order to discriminate among models (see Material and Methods).

**Figure 4:**
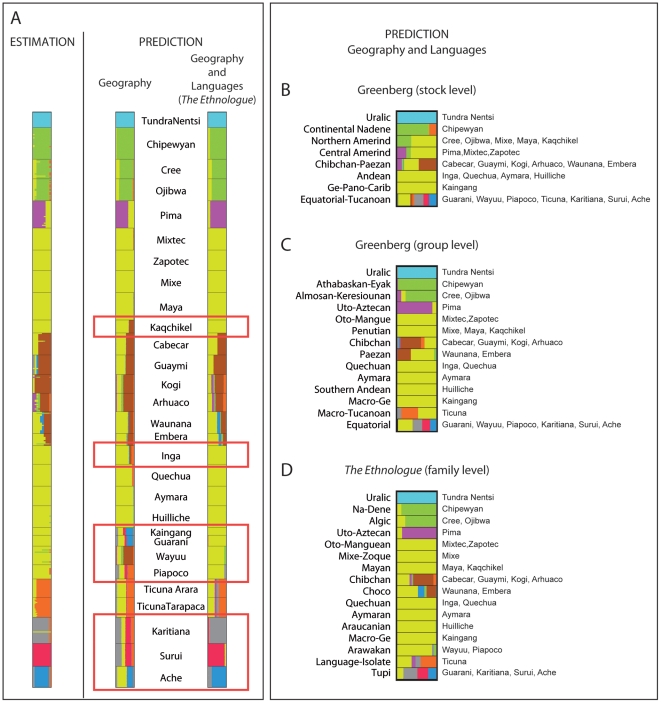
Estimated and predicted population genetic structure for 28 Native American populations. A. The membership coefficients are estimated in a model that includes spatial information (longitude, latitude). Inference of genetic structure is unchanged when we include additional linguistic covariates (Supporting Information Figure S1). The main differences between predictions obtained with or without linguistic information are framed in red. B-D. Membership coefficients predicted by Models B–D. The membership coefficients are averaged over individuals within the same linguistic unit.

The four clustering models resulted in highly similar patterns of estimated membership coefficients, and these patterns were also similar to the pattern found with structure (Figure 4, Figure S1, Wang *et al.* [15]). As we used a large number of microsatellite loci, these results are not surprising, and they warrant that the predictive power of the three linguistic classifications will be ascertained consistently.

**Figure 5:**
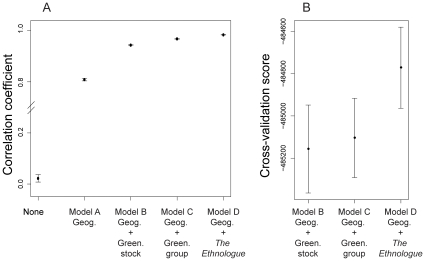
Variable selection for the Native American HGDP data. Geographic information includes longitude and latitude. Green. stands for Greenberg and Geog. stands for geography. The best model uses *The Ethnologue* linguistic classification.

**Figure 6:**
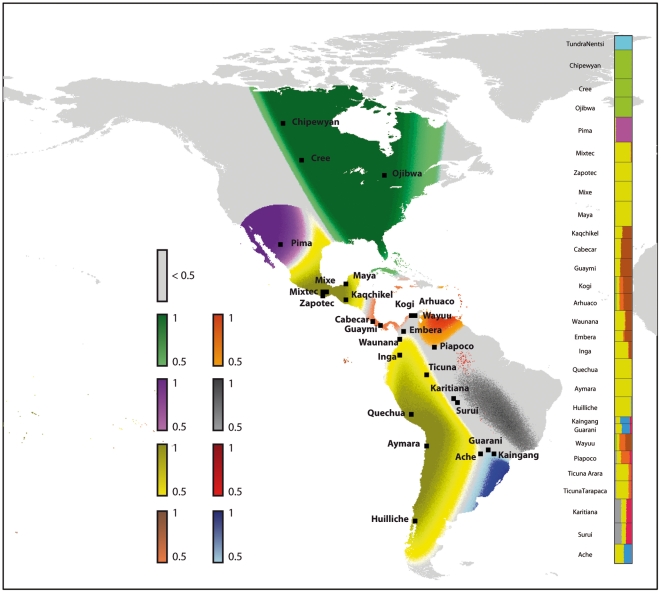
Genetic structure of Native American populations as predicted by geographical covariates. Geographical covariates include latitude, longitude, quadratic terms and an interaction term. Locations for which there is a cluster with a predicted membership coefficient larger than 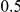 are colored with the cluster color. Locations for which there is no cluster that reaches the 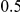 threshold or that are too distant from a sampled population are colored in grey. The barplot displays the membership probabilities as predicted by geographical covariates.

Using a quadratic trend surface to correct for geographic effects, we compared the predictions of a model without languages (Model A) to the predictions of a model using Greenberg's classification at the stock level (Model B), a model using Greenberg's classification at the group level (Model C), and a model using *The Ethnologue* classification (Model D). Figure 4A compares the predictions of Model A and Model D. For many population samples, the membership probabilities predicted by Model A were close to the estimated coefficients (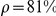, Figure 5A). The predictions of Model A for every geographic location in the American mainland are displayed in Figure 6. The value of the correlation coefficient and the map of predicted membership coefficients confirmed that geography is a good predictor of genetic structure in Native American populations. When including linguistic covariates (Models B–D), the predictions of cluster membership were closer to the estimates of the MCMC algorithm than those obtained without languages (Model A) except for the Pima. The correlation coefficient increased from 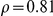 to 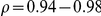 (Figure 5A), and the predicted genetic structure changed substantially (Figure 4A and Figure S1). For several populations the predictions obtained from linguistic covariates (Models B–D) differed from the predictions obtained with the geographic covariates only: Model A predicted that the Kaqchikel and the Wayuu samples shared substantial ancestry with a group comprising Cabecar, Guaymi, Kogi, Arhuaco, Waunana and Embera populations; Model A also predicted that the Kaingang and Guarani samples clustered with the Ache population, and that the Inga and Piapoco samples were grouped with the Ticuna sample.

Figure 4 B–D displays the membership coefficients predicted by POPS using Greenberg's and *The Ethnologue* classifications (Models B–D), grouping populations with the same linguistic taxon. At the exception of the Andean and Ge-Pano-Carib stocks, Greenberg's linguistic stocks were associated with multiple clusters (Figure 4B). Refining Greenberg's classification at the group level improved the characterization of genetic clusters by linguistic taxa (Model C, Figure 4C). At the group level, the Northern Amerind stock split into Almosan-Keresiouan and Penutian groups that correspond to genetically divergent clusters. Similarly, the Central Amerind stock split into Uto-Aztecan and Oto-Mangue groups which are also genetically divergent. However, the split of the Equatorial-Tucanoan stock into the Macro-Tucanoan and Equatorial groups, and the split of the Chibchan-Paezan stock into the Chibchan and Paezan groups, did not improve the prediction of genetic clusters. In *The Ethnologue* classification (Model D), the Equatorial group split into the Arawakan and Tupi families. This separation improved the prediction of genetic clusters since the Arawakan family was associated with a unique genetic cluster. In contrast, the separation of the Penutian group into the Mixe-Zoque and Mayan families did not improve the characterization of genetic groups. Overall *The Ethnologue* classification provided better predictions of genetic groups than Greenberg's classification. Among the 16 families of *The Ethnologue* classification, only the Tupi, Choco and Chibchan families were not associated to a unique genetic cluster (Figure 4D). Supporting these comparisons, Figure 5B shows that the cross-validation score increases when using *The Ethnologue* (Model D). The values of the cross-validation scores are approximately equal to 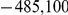 for Models B and C, and around 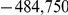 for Model D. These scores provide quantitative evidence that the classification of *The Ethnologue* leads to better predictions of genetic structure than Greenberg's classification at the stock or group levels.

## Discussion

We proposed a Bayesian latent class regression model to investigate to which extent geographic and linguistic information can predict population genetic structure in Native American populations. The originality of this approach was to model individual responses, i.e., the unobserved genetic cluster labels for each individual, using spatial and linguistic variables.

Our simulation study provided evidence that a hidden regression layer can improve the inference of genetic structure in addition to allowing their predictions from covariates. We also tested two criteria of variable selection based on correlation coefficients and cross-validation scores and found that these statistical indices reached a plateau when the true set of covariates was included in the POPS model. With small numbers of loci, the use of covariates decreased the misclassification rates of the clustering program significantly. For large numbers of loci, the estimation performances were hard to improve, especially when the likelihood dominated the prior distribution. However, using large numbers of loci made predictions and the use of the variable selection criteria reliable.

Using 678 microsatellite markers from the HGDP data set, we evaluated the suitability of geographic and linguistic predictors for Native American population genetic structure. Geography predicted genetic clusters rather accurately. However considering linguistic origin in addition to geographic origin improved the prediction of genetic structure. After correcting for geographic effects, we evaluated the predictive capabilities of three linguistic classifications: Greenberg's classification at two distinct levels and *The Ethnologue* classification. We did not consider Greenberg's tripartite classification (Amerind, Na-Dene, and Eskimo-Aleut) because, in addition to being controversial [48], all Native American HGDP populations, except the Chipewyan, belong to the Amerind family. We rather focused our analysis on taxonomically lower levels of Greenberg's classification: linguistic stocks and groups. Considering those refined levels, *The Ethnologue* provided better predictions of population genetic structure than Greenberg's classification.

Though *The Ethnologue* classification provided a better genetic proxy than Greenberg's classification, some linguistic families were not perfectly characterized in terms of genetic clustering. The Chibchan and Choco families were grouped in a Chibchan-Paezan stock by Greenberg [28]. These populations shared genetic ancestry with northern Mesoamerican populations (Mixtec, Zapotec, Mixe, Maya and Kaqchikel) and with southern Andean populations (Inga, Quechua, Aymara and Huilliche) (Figure 4A). Based on mtDNA data, Melton *et al.* [49] also found genetic relationships between Chibchan speakers and a Mayan population from Mesoamerica. To explain these relationships, it has been argued that Chibchan and Mesoamerican languages were all interrelated at one time into a larger Proto-Mesoamerican linguistic group that subsequently splintered into different language families after the intensification of agriculture in Mesoamerica [50], [51]. The shared genetic relationships between Mesoamerican populations and Chibchan-Choco populations would result from their shared common history. Another family lacking genetic characterization was the Tupi. The Tupi family encompasses approximately 41 languages that spread throughout eastern South America several millennia ago [52], [53]. Since the Tupi expansion involved language replacement, it may have blurred the relationships between genes and languages. Additionally, the Surui and Ache are populations with Tupi languages and small effective population sizes [15]. The ‘genetic patchwork’ of the Tupi would then result from genetic drift essentially.

Despite the intrinsic difference between methods, our analysis confirmed previous findings that a sizeable correspondence between genetic and linguistic differentiation may exist only below a certain level of linguistic differentiation. The tests of treeness indicated that language classifications provide the best fit to mitochondrial data when they included external features of language classification trees and no deeper internal relationships between languages [14]. Using partial Mantel tests, Wang *et al.* [15] found a low partial correlation (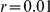) between linguistic (Greenberg's stock level) and genetic dissimilarities, but the correlation increased to 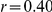 when the authors considered pairs of populations within stocks. Our analysis revealed that the congruence between genetic and linguistic diversification is more evident when considering a finer grain of linguistic differentiation than the stock level.

To further investigate potential scale effects, we applied POPS to 77 world-wide population samples from the HGDP data set excluding two language isolates (Basque and Burushaski) and grouping the sub-Saharian samples in a reference cluster (Table S2). The genetic clusters detected by POPS agreed with those detected by structure (Figure S2) [15], [54]. The geographic predictions of a quadratic trend surface model were highly correlated to the estimated membership coefficients (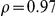). The high value of the correlation coefficient confirmed that geography is a good predictor of genetic structure at the world-wide scale [55]–[61]. Adding the linguistic covariates taken from *The Ethnologue* classification increased the correlation coefficient from 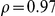 to 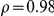. Thus it improved the prediction of genetic structure only marginally. These results provided evidence that the effects of language on the prediction of genetic structure are dependent on the scale considered. The results of POPS were also comparable to those obtained by Belle and Barbujani [23] reporting that languages have a small effect on the pattern of molecular variation at the world-wide scale. At the global scale, the patterns of genetic population structure are likely to reflect ancient demographic events, such as population divergence associated with the colonization of major geographic regions of the world [25]. At the continental scale, cultural traits contribute to the mediation of gene flow between human groups [62]. The predictive power provided by languages in the Americas could thus result from preferential mating within linguistic groups.

The examination of linguistic and genetic relationships in the Americas would obviously benefit from a more extensive sampling from the Na-Dene linguistic stock and from the inclusion of the Eskimo-Aleut stock. In a regression framework, a large dispersion of the explanatory variables is preferable. Though the sampling design of the HGDP was not optimal in our framework, our approach provided evidence that linguistic proxies improved the prediction of Native American population genetic structure. As human genomic data expand in genetic and geographic coverage [61], [63], [64], the use of latent class regression models could result in a more detailed picture of the role of geography and cultural factors in shaping human genetic variation.

## Supporting Information

Figure S1Estimated and predicted genetic structure of Native American populations, with 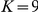 clusters, using different set of covariates in the probit model (Model A–D).(TIF)

Figure S2 Genetic structure at a worldwide scale as predicted by geographical covariates when K = 7. Geographical covariates include latitude, longitude and distance to the Addis Abeba, which is computed by included five obligatory waypoints. The three barplots correspond to 1) the genetic structure as inferred with genetic data and both spatial and linguistic covariates, 2) the structure as predicted with spatial information and 3) the structure as predicted with spatial and linguistic information. The linguistic variable is a qualitative variable corresponding to The Ethnologue classification.
(TIF)

Table S1 Coordinates and linguistic entities of 28 Native American populations from the Human Genome Diversity Panel.
(PDF)

Table S2 Coordinates, distance to Addis-Abeba, and linguistic families of 77 worldwide populations from the Human Genome Diversity Panel.
(PDF)

Appendix S1 Gibbs sampler.
(PDF)

Appendix S2 Computation of the predictive score for cross-validation.
(PDF)
