# Supplementary figures and images for: Predictions of Native American Population Structure Using Linguistic Covariates in a Hidden Regression Framework
Source: PLoS One. 2011 Jan 31;6(1):e16227. doi: 10.1371/journal.pone.0016227 (PMC3031544; doi:10.1371/journal.pone.0016227)

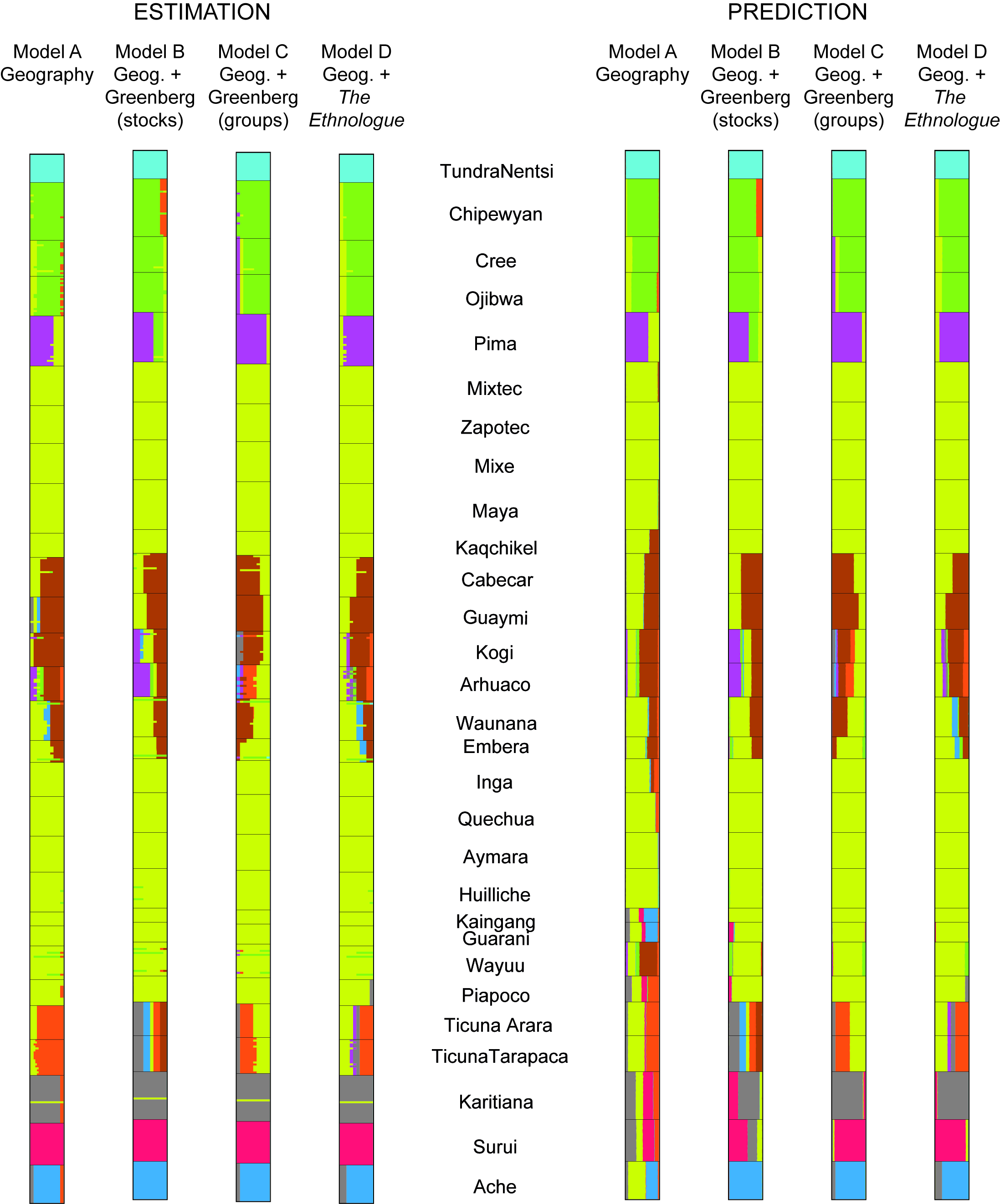

Supplement: Figure S1 — Estimated and predicted genetic structure of Native American populations, with clusters, using different set of covariates in the probit model (Model A–D). (TIF) [file pone.0016227.s001.tif]

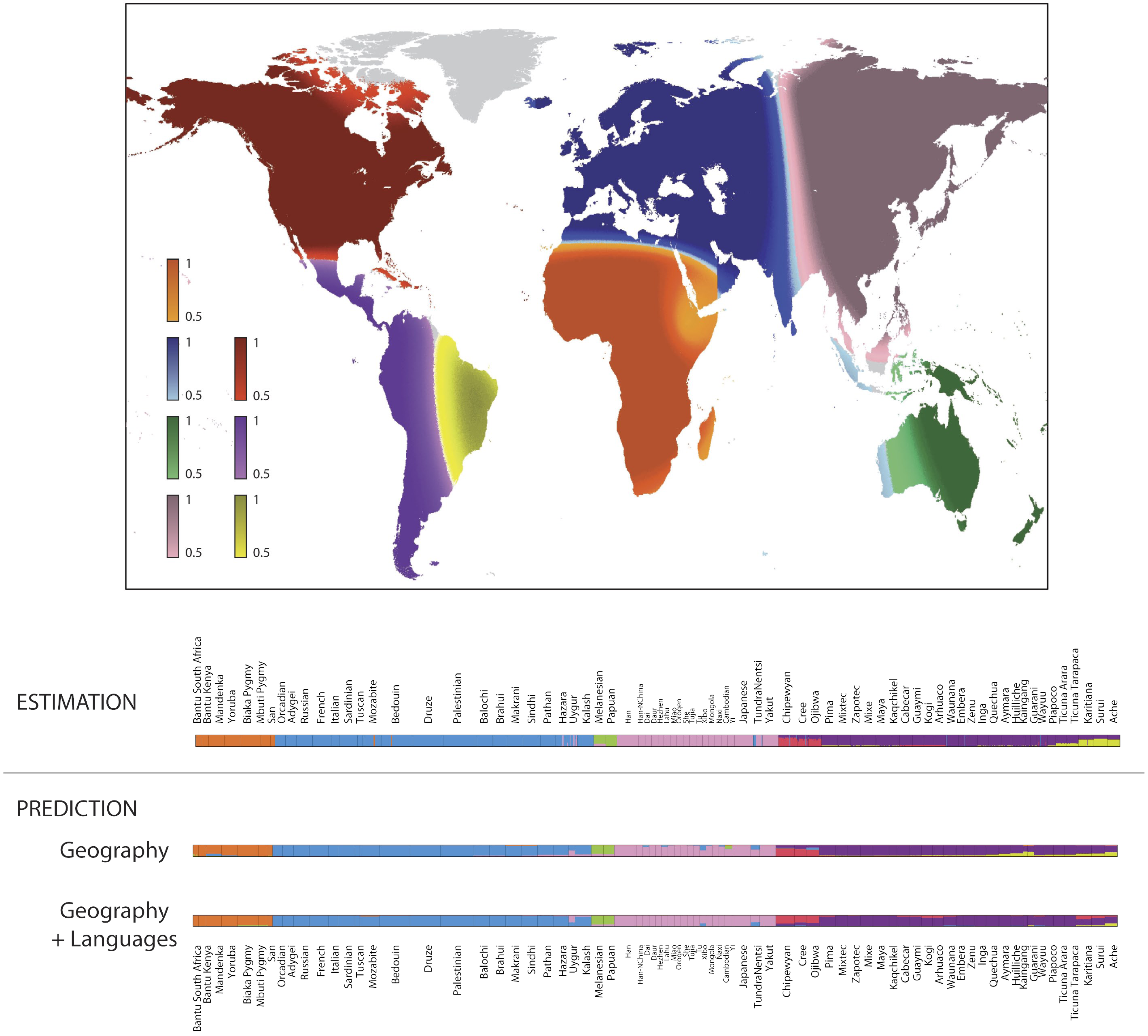

Supplement: Figure S2 — Genetic structure at a worldwide scale as predicted by geographical covariates when K = 7. Geographical covariates include latitude, longitude and distance to the Addis Abeba, which is computed by included five obligatory waypoints. The three barplots correspond to 1) the genetic structure as inferred with genetic data and both spatial and linguistic covariates, 2) the structure as predicted with spatial information and 3) the structure as predicted with spatial and linguistic information. The linguistic variable is a qualitative variable corresponding to The Ethnologue classification. (TIF) [file pone.0016227.s002.tif]
