# Supplementary material for: Predictions of Native American Population Structure Using Linguistic Covariates in a Hidden Regression Framework
Source: PLoS One. 2011 Jan 31;6(1):e16227. doi: 10.1371/journal.pone.0016227 (PMC3031544; doi:10.1371/journal.pone.0016227)
Supplement: Table S1 — Coordinates and linguistic entities of 28 Native American populations from the Human Genome Diversity Panel. (PDF) [file pone.0016227.s003.pdf]

Table : Coordinates and linguistic entities of 28 Native American populations from the Human Genome Diversity Panel.

Population	Latitude	Longitude	Green.[a] stock	Green.[a] group	Linguistic family (*The Ethnologue*)
Chipewyan	59.55	-107.3	Continental Na-Dene	Athabaskan-Eyak	Na-Dene
Cree	50.33	-102.5	Northern Amerind	Almosan-Keresiouan	Algic
Ojibwa	46.5	-81	Northern Amerind	Almosan-Keresiouan	Algic
Mixe	17	-96	Northern Amerind	Penutian	Mixe-Zoque
Maya	19	-91	Northern Amerind	Penutian	Mayan
Kaqchikel	15	-91	Northern Amerind	Penutian	Mayan
Pima	29	-108	Central Amerind	Uto-Aztecan	Uto-Aztecan
Mixtec	17	-97	Central Amerind	Oto-Mangue	Oto-Manguean
Zapotec	16	-97	Central Amerind	Oto-Mangue	Oto-Manguean
Cabecar	9.5	-84	Chibchan-Paezan	Chibchan	Chibchan
Guaymi	8.5	-82	Chibchan-Paezan	Chibchan	Chibchan
Kogi	11	-74	Chibchan-Paezan	Chibchan	Chibchan
Arhuaco	11	-73.8	Chibchan-Paezan	Chibchan	Chibchan
Waunana	5	-77	Chibchan-Paezan	Paezan	Choco
Embera	7	-76	Chibchan-Paezan	Paezan	Choco
Inga	1	-77	Andean	Quechua	Quechuan
Quechua	-14	-74	Andean	Quechua	Quechuan
Aymara	-22	-70	Andean	Aymara	Aymaran
Huilliche	-41	-73	Andean	Southern	Araucanian
Kaingang	-24	-52.5	Ge-Pano-Carib	Macro-Ge	Macro-Ge
Wayuu	11	-73	Equatorial-Tucanoan	Equatorial	Arawakan
Piapoco	3	-68	Equatorial-Tucanoan	Equatorial	Arawakan
Guarani	-23	-54	Equatorial-Tucanoan	Equatorial	Tupi
Karitiana	-10	-63	Equatorial-Tucanoan	Equatorial	Tupi
Surui	-11	-62	Equatorial-Tucanoan	Equatorial	Tupi
Ache	-24	-56	Equatorial-Tucanoan	Equatorial	Tupi
Ticuna Tarapaca	-4	-70	Equatorial-Tucanoan	Macro-Tucanoan	Language isolate
Ticuna Arara	-4	-70	Equatorial-Tucanoan	Macro-Tucanoan	Language isolate

[a] Green. stands for Greenberg
