# Supplementary material for: Predictions of Native American Population Structure Using Linguistic Covariates in a Hidden Regression Framework
Source: PLoS One. 2011 Jan 31;6(1):e16227. doi: 10.1371/journal.pone.0016227 (PMC3031544; doi:10.1371/journal.pone.0016227)
Supplement: Table S2 — Coordinates, distance to Addis-Abeba, and linguistic families of 77 worldwide populations from the Human Genome Diversity Panel. (PDF) [file pone.0016227.s004.pdf]

Table : Coordinates, distance to Addis-Abeba, and linguistic families of 77 worldwide populations from the Human Genome Diversity Panel.

Population	Latitude	Longitude	Distance to Addis-Abeba (km)	Linguistic family (*The Ethnologue*)
Bantu South East Africa	−28.40	27.6	4340	Niger-Congo
Bantu South West Africa	−21.00	18.7	4011	Niger-Congo
BantuKenya	−3.00	37.0	1354	Niger-Congo
Mandenka	12.00	−12.0	5585	Niger-Congo
Yoruba	8.00	5.0	3744	Niger-Congo
Biaka Pygmy	4.00	17.0	2495	Niger-Congo
Mbuti Pygmy	1.00	29.0	1422	Niger-Congo
San	−21.00	20.0	3934	Khoisan
Orcadian	59.00	−3.0	6189	Indo-European
Adygei	44.00	39.0	2990	North-Caucasian
Russian	61.00	40.0	4821	Indo-European
French	46.00	2.0	5524	Indo-European
Italian	46.00	10.0	4917	Indo-European
Sardinian	40.00	9.0	4896	Indo-European
Tuscan	43.00	11.0	4768	Indo-European
Mozabite	32.00	3.0	4507	Afro-Asiatic
Bedouin	31.00	35.0	2484	Afro-Asiatic
Druze	32.00	35.0	2594	Afro-Asiatic
Palestinian	32.00	35.0	2594	Afro-Asiatic
Balochi	30.50	66.5	3835	Indo-European
Brahui	30.50	66.5	3835	Dravidian
Makrani	26.00	64.0	3775	Indo-European
Sindhi	25.50	69.0	4251	Indo-European
Pathan	33.50	70.5	4152	Sino-Tibetan
Hazara	33.50	70.0	4105	Indo-European
Uygur	44.00	81.0	5105	Altaic
Kalash	36.00	71.5	4227	Indo-European
Melanesian	−6.00	155.0	14030	Austronesian
Papuan	−4.00	143.0	12792	Austronesian
Han	32.50	114.0	8112	Sino-Tibetan
Han-NChina	39.00	114.0	7875	Sino-Tibetan
Dai	21.00	100.0	7338	Austronesian
Daur	48.50	124.0	8338	Altaic
Hezhen	47.50	133.5	9040	Altaic
Lahu	22.00	100.0	7289	Sino-Tibetan
Miao	28.00	109.0	7852	Hmong-Mien
Oroqen	50.50	126.5	8444	Altaic
She	27.00	119.0	8799	Hmong-Mien
Tujia	29.00	109.0	7810	Sino-Tibetan
Tu	36.00	101.0	6856	Altaic
Xibo	43.50	81.5	5137	Altaic
Yi	28.00	103.0	7304	Sino-Tibetan
Mongola	48.50	119.0	7982	Altaic
Naxi	26.00	100.0	7110	Sino-Tibetan
Cambodian	12.00	105.0	8289	Austro-Asiatic
Japanese	38.00	138.0	9838	Japonic
Tundra Nentsi	66.08	76.5	5956	Uralic
Yakut	63.00	129.5	8258	Altaic
Chipewyan	59.55	−107.3	10251	Na-Dene
Cree	50.33	−102.5	11182	Algic
Ojibwa	46.50	−81.0	12490	Algic
Pima	29.00	−108.0	12898	Uto-Aztecan
Mixtec	7.00	−97.0	15606	Oto-Manguean
Zapotec	16.00	−97.0	14698	Oto-Manguean
Mixe	17.00	−96.0	14644	Mixe-Zoque
Maya	19.00	−91.0	14679	Mayan
Kaqchikel	5.00	−91.0	16085	Mayan
Cabecar	9.50	−84.0	15959	Chibchan
Guaymi	8.50	−82.0	16154	Chibchan
Kogi	11.00	−74.0	16277	Chibchan
Arhuaco	11.00	−73.8	16286	Chibchan
Waunana	5.00	−77.0	16742	Choco
Embera	7.00	−76.0	16587	Choco
Zenu	9.00	−75.0	16432	Choco
Inga	1.00	−77.0	17145	Quechuan
Quechua	−14.00	−74.0	18797	Quechuan
Aymara	−22.00	−70.0	19782	Aymaran
Huilliche	−41.00	−73.0	21482	Araucanian
Kaingang	−24.00	−52.5	20797	Macro-Ge
Guarani	−23.00	−54.0	20629	Tupi
Wayuu	11.00	−73.0	16323	Arawakan
Piapoco	3.00	−68.0	17363	Arawakan
Ticuna Tarapaca	−4.00	−70.0	17978	Language-Isolate
Ticuna Arara	−4.00	−70.0	17978	Language-Isolate
Karitiana	−10.00	−63.0	18910	Tupi
Surui	−11.00	−62.0	19057	Tupi
Ache	−24.00	−56.0	20636	Tupi
