# Supplementary material for: Predictions of Native American Population Structure Using Linguistic Covariates in a Hidden Regression Framework
Source: PLoS One. 2011 Jan 31;6(1):e16227. doi: 10.1371/journal.pone.0016227 (PMC3031544; doi:10.1371/journal.pone.0016227)
Supplement: Appendix S1 — Gibbs sampler. (PDF) [file pone.0016227.s005.pdf]

APPENDIX S1: GIBBS SAMPLER

To sample from the posterior distribution of the cluster labels Z, the allelic frequencies P and the regression coefficients β, we implemented a Markov Chain Monte Carlo algorithm with Gibbs sampling steps.

UPDATING P. This step is the same as in the software `structure`. It is performed by simulating the set of frequencies as

$$p_{kl.}|X, Z \sim \mathcal{D}(\lambda + n_{kl1}, \ldots, \lambda + n_{klJ_l}), \tag{1}$$

where $p_{kl.}$ denotes the vector of allele frequencies in the cluster k at the locus l, and n_{klj} denotes the number of copies of the allele j in population k at the locus l, $k = 1, \ldots, K$, $l = 1, \ldots, L$, $j = 1, \ldots, J_l$. For our analysis, we considered $\lambda = 1$.

UPDATING (W, Z). Since Z can be obtained from W in a deterministic fashion, Z and W are updated simultaneously. Using the Bayes formula, the joint conditional distribution of (W, Z) can be written as

$$\Pr(W, Z|\beta, P, X) \propto \Pr(X|\beta, P, Z)\Pr(W|\beta)\Pr(Z|W)$$

To simulate the couples (W, Z), we use the following rejection algorithm.

- Step 1. For $i = 1, \ldots, n$, simulate the couple (W_i, Z_i) from the multinomial probit model by generating W_i from regression equation and determine $Z_i = k$ with its max-rule (see Methods, equations (1) and (2)).
- Step 2. Accept the couple (W_i, Z_i) with probability

  $$\frac{\Pr(X_i = x_i|P, Z_i = k)}{\max_k \Pr(X_i = x_i|P, Z_i = k)},$$

  and return to step 1. The likelihood function $\Pr(X_i = x_i|P, Z_i = k)$ is given by equation (2) in [1].

UPDATING BETA We choose a noninformative prior distribution for β, $\beta \sim \mathcal{N}(0, A^{-1})$, with $A = 0$. The Gibbs

sampler proceeds by updating values of β using its conditional distribution [2]

$$\beta | W \sim \mathcal{N}(V\tilde{X}^T W,\ V), \text{ where } V = (\tilde{X}^T\tilde{X})^{-1}. \tag{2}$$

REFERENCES

1. Pritchard JK, Stephens M, Donnelly P (2000) Inference of population structure using multilocus genotype data. Genetics 155: 945-959.

2. Albert JH, Chib S (1993) Bayesian analysis of binary and polychotomous response data. J Am Stat Assoc 88: 669–679.
