# Supplementary material for: Predictions of Native American Population Structure Using Linguistic Covariates in a Hidden Regression Framework
Source: PLoS One. 2011 Jan 31;6(1):e16227. doi: 10.1371/journal.pone.0016227 (PMC3031544; doi:10.1371/journal.pone.0016227)
Supplement: Appendix S2 — Computation of the predictive score for cross-validation. (PDF) [file pone.0016227.s006.pdf]

APPENDIX S2: COMPUTATION OF THE PREDICTIVE SCORE FOR CROSS-VALIDATION

Let X^{training} be a subset of the loci used for inferring the parameters of the clustering model. The log-probability of the complementary set of loci $X^{\text{validation}}$ is a function of the cluster labels Z given by

$$\log(\Pr(X^{\text{validation}}|Z)) = \sum_{k=1}^{K} \sum_{l \in X^{\text{validation}}} \log \Pr(x_l^{[k]}) \tag{1}$$

where $x_l^{[k]}$ denote the observed genotypes at locus l in cluster k. We denote by $n_{kl} = (n_{kl1}, \ldots, n_{klJ_l})$ the allele count at locus l in cluster k. The allele counts follow a multinomial distribution $n_{kl} \sim$ Multinomial$(m_{kl}, p_{kl1}, \ldots, p_{klJ_l})$, where $m_{kl} = \sum_{j=1}^{J_l} n_{klj}$ is the number of different genotypes at locus l in cluster k . By integrating over the alleles frequencies, we find that

$$\begin{aligned} \Pr(x_l^{[k]}) &= \frac{\prod_j (n_{klj}!)}{m_{kl}!} \Pr(n_{kl}|\lambda) \\ &= \frac{\Gamma(\lambda J_l)}{\Gamma(m_{kl} + \lambda J_l)} \prod_j \frac{\Gamma(n_{klj} + \lambda)}{\Gamma(\lambda)}. \end{aligned}$$

We computed the predictive score after averaging the quantities in equation (1) over the posterior distribution of the cluster labels Z given by the training data set.
